# Multiple policy approaches in improving community pharmacy practice: the case in Indonesia

**DOI:** 10.1186/s12913-018-3258-8

**Published:** 2018-06-14

**Authors:** Andi Hermansyah, Erica Sainsbury, Ines Krass

**Affiliations:** 1 Faculty of Pharmacy, The University of Sydney, A-15 Pharmacy and Bank Building, Sydney, 2006 New South Wales Australia; 2 Faculty of Pharmacy, Airlangga University, Jl. Dharmawangsa Dalam, Surabaya, 60286 East Java Indonesia

**Keywords:** Community pharmacy practice, Policy approaches, Policy evaluation, Indonesia

## Abstract

**Background:**

Health reform has been an ongoing agenda in many countries with community pharmacy increasingly gaining attention for contributing to healthcare improvement. Likewise, multiple policy approaches have been introduced to improve community pharmacy practice in Indonesia yet no studies have evaluated their effectiveness. Therefore, this study aimed to identify and collate information on approaches intended to improve practice in Indonesian community pharmacy and subsequently examine the perceptions of key stakeholders in healthcare and community pharmacy about these approaches and the extent to which they have affected community pharmacists as a profession.

**Methods:**

This study reviewed the grey literature related to community pharmacy policies published by government and pharmacy organisations in Indonesia since 2009 and broadened the search to other relevant databases. In-depth semi structured interviews were conducted with a wide range of key stakeholders in pharmacy and healthcare between February and August 2016 to evaluate these policy approaches.

**Results:**

Seventeen policy documents were identified with the majority published by the Indonesian Pharmacists’ Association (8 documents) and Ministry of Health of Indonesia (6 documents). Most documents (15 documents), either the updated version or new policy, were published since 2014 indicating the recent enthusiasm of pharmacy stakeholders to improve community pharmacy practice. Twenty-nine key stakeholders participated in the study, and highlighted three main themes regarding the policy approaches: barriers to effective policy implementation, need for policy changes and strategies to cope with policy challenges. Poor policy enforcement was commonly expressed by participants as a major challenge, with participants anticipating the need for a unified stakeholder vision to improve the current situation. Participants also mentioned several local initiatives which they claimed were improving practice but evidence was lacking.

**Conclusion:**

The introduction of policy initiatives within the past ten years has highlighted the enthusiasm of policy makers and pharmacy stakeholders to improve community pharmacy practice in Indonesia. However, some of the initiatives were conceived and enacted in a piecemeal, sometimes conflicting and uncoordinated way. Overall, fundamental and entrenched barriers to practice need to be overcome to create a more professional climate for the practice of pharmacy in Indonesia.

**Electronic supplementary material:**

The online version of this article (10.1186/s12913-018-3258-8) contains supplementary material, which is available to authorized users.

## Background

Governments around the world face increasing pressure to provide effective, efficient and equitable healthcare services to their populations. Health reform has been on the main agenda in many countries with similar approaches applied to improve access to health care and the overall performance of the health system within the constraints of needing to curb the growth in health expenditure [1]. One reform that is increasingly gaining attention is to incorporate community pharmacists within the broader healthcare system. Community pharmacists have the potential to not only contribute to improving patients’ outcomes through safe and effective use of drugs, but also to reduce the cost of healthcare by resolving drug related problems and promoting public health issues [2, 3].

At the same time, the nature of pharmacy practice and community pharmacy is also changing. Over the past four decades, scholars have acknowledged a shift in community pharmacy practice beyond dispensing activities to provision of a broader array of health services [4–6]. These simultaneous changes have resulted in complexity for all stakeholders, requiring them to adapt to rapidly evolving circumstances. As a result, community pharmacists have consistently been challenged with pressures to meet professional standards, to provide patient-centered services, and to work with other healthcare professionals within the large healthcare system while keeping profitable in a highly-regulated environment.

Policy makers also face complexity in attempting to encompass both an increase in the utilization of community pharmacists, while maintaining some control over the increasing health care budget. Policy documents since the Nuffield report in 1986 [7], including more recent blue prints or road maps for the future of pharmacy, highlight the multiple approaches and strategies that have been directed towards harnessing a greater contribution by pharmacy to health care [8–10]. Despite the growing number of initiatives to improve the practice of community pharmacy, the literature on policy evaluation is sparse. In addition, much less attention has been directed to determining how stakeholders in community pharmacy perceive the impact of these policy statements and initiatives on pharmacy practice. Furthermore, policy development in expanding the role of community pharmacists has not always been supported by relevant policy evidence which in turn has raised questions about the extent to which these policies have been appropriate, effective and sustainable particularly for stakeholders in community pharmacy [4].

The lack of policy evaluation has been common in both developed and developing countries. However, the situation is arguably more acute in developing countries. There is limited capacity among stakeholders, particularly government, to fund and produce quality research which examines the practice of community pharmacists and pharmacy [11]. Furthermore, regulatory evaluation is also constrained by a myriad of factors encompassing limited government staff, small budgets, fragmented delivery of healthcare and pharmacy practice, poor control over the regulation and the absence of a regulatory evaluation framework which may be less pronounced in developed countries [12]. Thus, there is an urgent need to evaluate the impact of various policy or program initiatives designed to influence pharmacy practice in developing countries. This paper aims to address this using Indonesian community pharmacy as a case study.

The Indonesian health system has undergone significant changes over the past decade including the establishment of a decentralization policy in 2001 and the recent introduction of universal healthcare coverage (JKN) in 2014 [13]. With respect to community pharmacy, multiple approaches and regulations intended to advance the practice of community pharmacy have been enacted within the past decade. These approaches include legislation, incentivization policies, campaigns and education [14]. Prior to critically examining the effectiveness of these multiple approaches, it is important to contextualize the policy and practice environment in Indonesia in order to understand the nature of the system and challenges to implementation.

### Policy environment of Indonesian community pharmacy sector

Community pharmacy practice in Indonesia is regulated under the Ministry of Health (MoH) at the national level, and the Local Health Department office as the extension of MoH at the provincial and district (Kabupaten/Kota) level. In addition, the 2001 decentralization policy transferred the responsibility for services delivery and fiscal autonomy, including health, from the central government to the local government. At the same time, the operation of community pharmacy is supervised by BPOM (Indonesia National Agency of Drug and Food Control) as the government agency responsible for the administration and control of food and drugs. Further, the authority for overseeing drugs and therapeutic products in the market is also part of the duty of the police. The police with or without BPOM often conduct surprise inspections of healthcare facilities including pharmacy particularly when they suspect illegal activities such as selling of prescribed medicines without a doctor’s prescription, selling of expired medicines and selling of unlicensed medicines. Importantly, it should be noted that there is no legal restriction preventing pharmacists who work in regulatory or supervisory bodies from also practicing in community pharmacy, despite the potential for a conflict of interest to arise. However, the chairman of BPOM recently issued a directive to BPOM staff prohibiting them from working in any facilities under the supervision of BPOM including community pharmacy [15]. As a result, the majority of the staff have resigned from their employment in any pharmacy settings in order to retain their position in BPOM [16].

From the professional practice point of view, the Indonesian Pharmacists Association (IAI) is the sole peak pharmacy organization representing pharmacists, with the main role being to maintain pharmacists’ competence, advocate for pharmacists and advance the profession. Within the IAI, there are several peer groups based on work setting and professional interest including a group of community pharmacists (HISFARMA) which is responsible for coordinating and advancing the practice of pharmacists in the community as defined by IAI. Another important body is the Indonesian Pharmaceutical Association (GP Farmasi) whose membership includes business owners in the pharmacy sector comprising pharmaceutical industries, wholesalers, pharmacies and retail drug outlets. Since the ownership of community pharmacy in Indonesia is not restricted to pharmacists, members of GP Farmasi representing community pharmacy include non-pharmacists. Responding to the introduction of JKN in 2014, both IAI and GP Farmasi worked together to establish a community pharmacy association (ASAPIN) as an organization to represent the whole community pharmacy network in the negotiation of tariffs within JKN. However, despite its vital mission, the MoH to date has not included ASAPIN in the legislation of healthcare facilities negotiating for JKN. Consequently, pharmacists and the community pharmacy network are underrepresented in the JKN payment scheme. Another organization which has a role in determining the quality and competence of pharmacists is the National Pharmacy Board (KFN) which manages the registration of pharmacists and oversees the Association of Schools of Pharmacy (APTFI) which deals with the development of pharmacy education curricula and competence of graduates.

The foregoing discussion has highlighted the complexity of the management and oversight of community pharmacy and pharmacists in Indonesia. Multiple regulators and professional organisations play overlapping, and sometimes conflicting roles in influencing the practice of pharmacy, and the way it is evolving. Each of these institutions has advocated top-down policies, standards and legislation which are parallel to the mission of other institutions. Therefore, the first objective of the current study was to identify and collate information on initiatives intended to improve practice in Indonesian community pharmacy. The second objective was to examine the perceptions of key stakeholders in community pharmacy about the multiple approaches advocated by the government and pharmacy organisations, and the extent to which these approaches have affected community pharmacists as a profession and resulted in practice change.

## Methods

### Document collection and analysis

We searched websites of relevant government departments and professional organisations in particular the websites of the Ministry of Health of Indonesia, BPOM, IAI, GP Farmasi, APTFI and KFN. These websites were searched for relevant information, reference publications and databases describing pharmacists’ role and responsibility and community pharmacy practice. The search process was broadened to include grey literature obtained from databases of other government institutions. As most government documents and professional organizations’ policies were published in the Indonesian language, this study used the following combination of search terms in the Indonesian language: apotek OR apotik OR farmasi (meaning: community pharmacy); apoteker OR farmasis OR tenaga AND farmasi (meaning: pharmacists); praktek OR praktik OR pekerjaan OR pelayanan AND farmasi (meaning: pharmacy practice or pharmacy services); kebijakan OR peraturan OR hukum OR keputusan OR standar OR pedoman OR rencana AND farmasi (meaning: legislative framework in pharmacy); peran OR kinerja OR tanggungjawab AND apoteker AND apotek OR apotik (roles and responsibility of pharmacists or pharmacy). In addition to the web search, investigators contacted key personnel in government agencies and professional organizations for further information/clarification and to request copies of relevant documents if necessary. This study purposively selected grey literature, and used an exploratory approach because there has been limited research published in the peer reviewed literature, in the field of policy evaluation of community pharmacy practice in Indonesia.

**Fig. 1:**
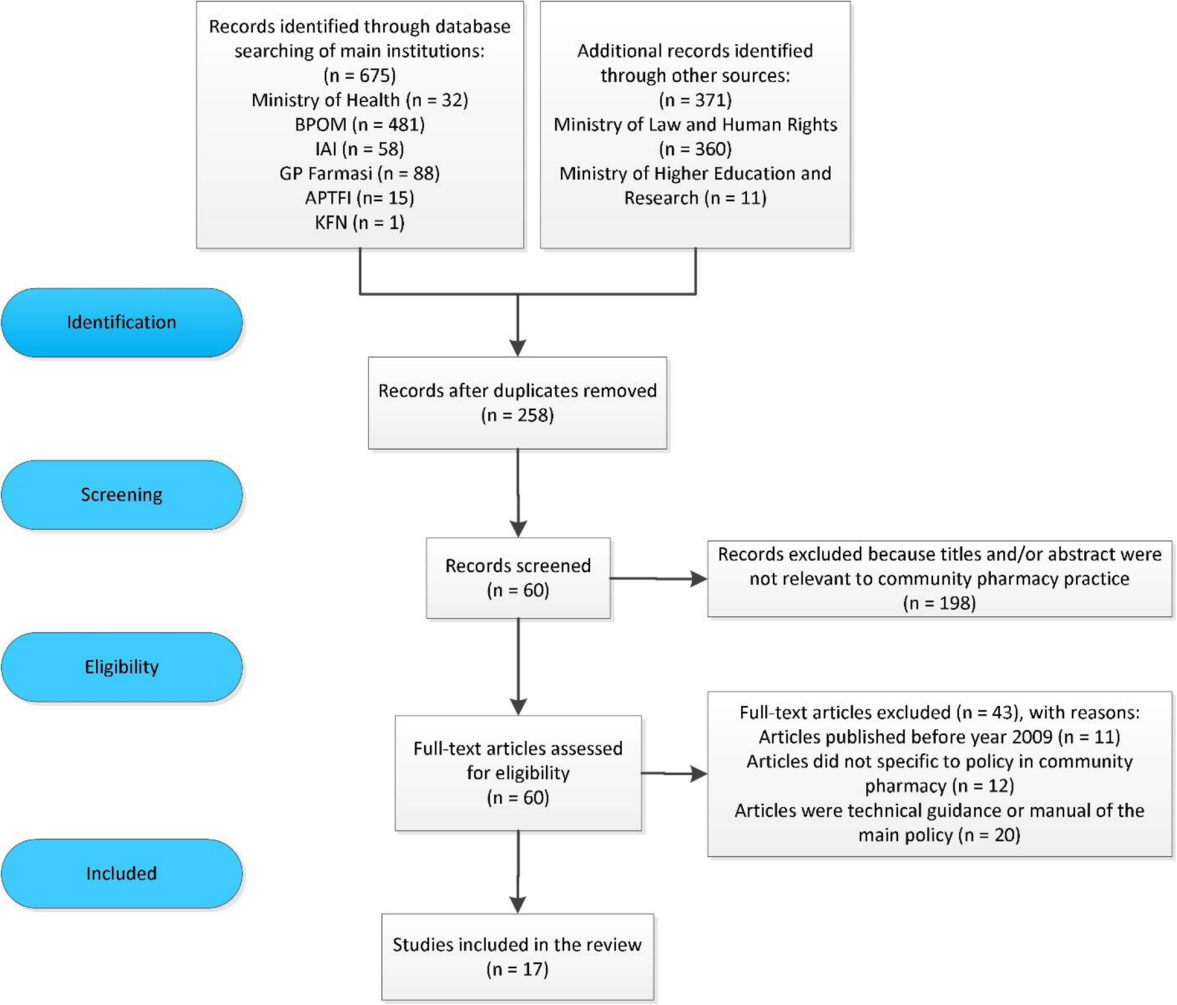
Flow diagram of articles selection process using PRISMA [28]

In terms of the inclusion and exclusion criteria, this study only included formal and legal documents from official authorities such as policy documents, legislative frameworks, standards and directives. We limited the search for documents published in the Indonesian language from 2009 onwards. The 2009 start date was selected because this was the year in which Health Law 2009 was issued. It was the first law accommodating the practice of pharmacy in Indonesia (article 108). The implication of this law was the enactment of the Pharmacy Practice Act 2009 which became the main policy framework underpinning the practice of pharmacy including community pharmacy in Indonesia. Details of literatures search and screening process are shown in Fig. 1.

The study retrieved 60 documents, of which 17 contained relevant information on policies and strategies aimed to improve pharmacy practice in Indonesia. The selected documents were reviewed based on their objectives and relevance to the support of community pharmacy practice either by: (1) providing a legal framework for practice, (2) reducing barriers in practice, (3) increasing the role and recognition of pharmacists, (4) promoting the uptake of pharmacy services, (5) contributing to the sustainability of community pharmacy operation. The criteria used to review the documents were developed from the recommendations of several systematic reviews reporting on pharmacy practice in developing countries [12, 17–20].

### Stakeholder collection and analysis

Between February and August 2016, in-depth semi structured interviews were conducted with a wide range of key stakeholders in pharmacy and healthcare representing community pharmacists, physicians, peak pharmacy and medical organisations, insurance companies, consumer group associations and governments in the local and national setting. Ethics approval was obtained from the authors institute prior to data collection. A purposive sampling was used to select the initial respondents and expanded using the snowball method. Candidates who agreed to participate were required to provide signed consent prior to the interview. The respondents were asked questions primarily focused on the current situation of healthcare and the community pharmacy sector, approaches introduced by governments and pharmacy associations to cope with the changes and challenges of the current situation, their expectations and strategies to adapt to the challenges (see Additional file 1). All interviews were audio-recorded and transcribed verbatim. Thematic analysis was performed to analyse the findings. Each investigator initially developed a coding framework from some of the transcripts which were considered unique and “rich of information”. Subsequently, the main coding framework which included themes and sub-themes was mutually agreed. The interviews were continued until data saturation was achieved. NVivo 10 was used to assist data management.

## Results

### Collation of documents

Of the 17 documents which were eligible for inclusion, the majority were published by IAI (8 documents) and the Ministry of Health of Indonesia (6 documents). The remainder were issued by KFN (1 document), the Presidential office (1 document) and a group of organisations – IAI, APTFI and KFN (1 document). According to the hierarchy of legislation in Indonesia, the Presidential Act is the highest level of legislation, followed by Ministry of Health decrees, and professional organizations’ regulations as subsidiary legislation, respectively. This means most of the approaches collated in this study were applied in a narrow setting and have limited legislative power. For instance, the initiative to set minimum remuneration for pharmacists enacted by several branches of IAI was only applicable to pharmacists within the region covered by the branches.

Most documents concerned approaches which were commenced from 2014 onwards (15 documents). However, some of these were updated versions of previous legislation (6 documents). This means that some approaches have changed over a long period of time. For example, the Community Pharmacy Decree was first introduced in 1953, and subsequently revised in 1965, 1980, 1993, 2002 and currently in 2017. It is also important to note that some approaches have certain degree of overlap with other approaches. For example, drug use campaign programs such as Gema Cermat, Dagusibu and GKSO were in essence devised to convey similar public educational messages surrounding self-management and basic education on the use of medicines although they were initiated by different organisations. As a result, community pharmacists may undertake one single public education activity and offer it as part of all three programs, thus gaining multiple CPD credits for the same activity. Some approaches also form part of the process of other policy initiatives. For example, pharmacists who wish to renew their practice license must deliver practice as defined by standard of pharmacy practice and pharmacy services, participate in CPD programs and collect a certain amount of credits. Table 1 is a compilation of the multiple approaches advocated by government and professional organisations in Indonesia.

**Table 1:** Multiple Approaches to improve community pharmacy operation and practice of pharmacists

Approaches (initiating bodies, year introduced/updated); References	Objective	Process to achieve objective
*Type of approaches: Incentivization*		
Minimum rates for pharmacists’ remuneration (branches of IAI^a^, introduced in 2015); [29–31]	Ensuring pharmacists receive adequate and fair income	Pharmacy employer must pay employee pharmacists based on the minimum rate. The amount and composition of the income must be validated by IAI and become the consideration for issuing recommendation letter for pharmacists to practice
Payment for pharmacy services (MoH, updated in 2016); [32]	Reimbursing pharmaceuticals and incentives for pharmacy services	Community pharmacies working under the JKN^b^ scheme receive payment for dispensing prescribed medicines and incentives for delivering pharmacy services. The method for distribution, the amount and the coverage of the payment can vary depending on the classification of pharmacy i.e. pharmacy affiliated with primary care providers, contracted by BPJS Health or both.
*Type of approaches: Campaigns and communication*		
Gema Cermat - Community awareness campaign in using medicines (MoH, introduced in 2015); [33]	Raising peoples’ awareness on proper use of medicines	National campaign including workshops, group discussions and distribution of information e.g. books, posters, modules and audio-videos. Certain pharmacies and community pharmacists are invited to deliver public education such as talks, lectures and community outreach.
Dagusibu - Pharmacists campaign on self-management of medicines (IAI, introduced in 2014); [34]	Public education on self-management of medicines	Community pharmacies are encouraged to provide educational materials such as leaflets, brochures and posters in the pharmacy. With the phrase “Ask your pharmacist”, consumers are educated to obtain, use, store and dispose of medicines as advised by pharmacists. Participating pharmacists are rewarded with credits (SKP^c^) for license renewal.
Gerakan Keluarga Sadar Obat (GKSO) - Campaign for raising family awareness in using medicines (IAI, introduced in 2014); [34]	Raising family awareness on self-management of medicines	Run in tandem with Dagusibu program, GKSO targets the health of family through lectures, simulation and role play, CBIA^d^ (active individual learning), training of pharmacists as trainers and recruitment of family members as health advocates. Topics for learning also include safe and proper use of cosmetics, food, beverages and narcotics/psychotropics. Participating pharmacists are rewarded with credits (SKP) to count towards their license renewal.
Image building of pharmacists (IAI, introduced in 2014); [35]	Increasing pharmacists’ recognition	Pharmacists are encouraged to wear pharmacist coat and name badge during practice in community pharmacy. The pharmacy must also display a sign board showing pharmacists’ names and practice hours. Credits (SKP) are awarded for pharmacist’s license renewal.
*Type of approaches: Standard, policy and regulation*		
Registration, certification and licensure of pharmacists (MoH, updated in 2016); [36]	Ensuring that pharmacists practice in a professional and ethical manner	Community pharmacists are required to obtain four legal documents to practice; certificate of competence and recommendation letter issued by IAI, registration letter (STRA^e^) from the National Board of Pharmacy (KFN^f^) and license to practice (SIPA^g^) from the MoH. In order to obtain certificate of competence, new graduate pharmacists must pass a competency exam while registered pharmacists must collect a quantum amount of credits (SKP) during each five years of practice. The certificate is a pre-requisite to obtain STRA. Once the STRA has been issued, pharmacists must apply for a recommendation letter. The letter of recommendation and STRA are part of the application for SIPA. The license is valid for five years and a pharmacist can practice in up to three different pharmacies. Prior to expiration, pharmacists must renew the license by firstly obtaining a new certificate of competence. The updated regulation has allowed pharmacists to practice in up to three community pharmacies.
Collection of SKP (IAI, introduced in 2014); [37]	Indicator for pharmacists’ participation in practice	Pharmacists must collect minimum of 150 credits (SKP) during each five years of practice as a requirement for license renewal. In general, the credits are distributed to participation in continuing education program (e.g. CPD, workshop and peer group discussion) minimum 60 credits, undertaking professional practice (indicated by attendance report and record of providing services) minimum 60 credits, involvement in community outreach program (e.g. public campaign) minimum 7.5 credits, and voluntary participation in publishing ideas and knowledge development (e.g. conducting research, writing book and article) maximum 37.5 credits.
Pharmacy Practice Act (MoH, introduced in 2009); [38]	Legislating pharmacy practice	The Act which underpins pharmacy practice in Indonesia regulates different settings of pharmacy practice from manufacturing and distribution to service provision including community pharmacy. It also classifies the pharmacy workforce into two main groups: pharmacists and pharmacy technicians, with their designated responsibilities. The act legislates that pharmacy practice can only be conducted under responsibility and supervision of pharmacists.
Standard of pharmacy services in community pharmacy (MoH, updated in 2016); [39]	Setting minimum services delivered in pharmacy	The standard describes two main roles conducted by community pharmacists: management of pharmaceuticals and healthcare devices, and provision of clinical pharmacy services. The first role relates to the management cycle of pharmacy items from planning and procurement to disposal, record keeping and reporting. The second role covers pharmacy services which should be provided by pharmacists such as prescription assessment, dispensing, drug information, counselling, home pharmacy care, drug use monitoring and surveillance for adverse drug reactions.
Standard for pharmacy practice (IAI, introduced in 2014); [40]	Developing standard for pharmacists to practice	The standard consists of 9 (nine) key activities which must be conducted during practice: (1) providing fundamental pharmacy practice, (2) conducting drug assessment and review, (3) dispensing medicines and health devices, (4) compounding dosage form (specific to pharmacists in the pharmaceutical industries), (5) providing drug information and counselling, (6) delivering health promotion, (7) management of pharmaceuticals and health devices, (8) management of pharmacy settings, (9) maintaining skills and competencies. The standard sets minimum activities for pharmacists in the practice site.
Standard competency of pharmacists (IAI-APTFI-KFN, updated in 2016); [41]	Setting the minimum competency of practicing pharmacists	The standard comprises 10 (ten) main competencies which means pharmacists must be competent in: (1) delivering the practice of pharmacy in an ethical and professional manner, (2) optimising the use of medicines, (3) dispensing medicines and health devices, (4) providing information about the medicines and health devices, (5) mastering skills and knowledge of formulation and production of pharmaceuticals, (6) contributing to preventive and promotive community health, (7) management of medicines and health devices, (8) delivering effective communication, (9) active involvement in the organization and maintaining inter-personal relationship, (10) striving to improve competency. Graduate pharmacists must meet the minimum competency as defined by the standard.
Community pharmacy Decree (MoH, updated in 2017); [42]	Establishing regulation for community pharmacy operation	The decree is the main framework regulating the opening, license issuance and operation of community pharmacy. A community pharmacy can be opened by pharmacists with or without investment from other parties (individual, group or organization). An approval from the MoH, which can be delegated to the district government, is required before opening a pharmacy. In addition, district government has the right to manage the location and distribution of community pharmacy. Premises, facilities, and equipment of the pharmacy must meet certain standards and be approved prior to operation. The practice of pharmacy must comply with the regulation as similarly stated in the Pharmacy Practice Act and Standard of Pharmacy Services in Community Pharmacy. Each pharmacy must have a First-pharmacist as pharmacist in-charge for the operation and practice of pharmacy who can be assisted by other pharmacists (as second-pharmacist), technician and/or administrative employee.
*Type of approaches: education and training*		
Continuing Professional Development (IAI, introduced in 2014); [43]	Improving pharmacists’ competence and knowledge	Pharmacists are encouraged to participate in CPD program. Pharmacists undertaking CPD program are rewarded with credits (SKP) which are essential for license renewal
Pharmacists Competency Examination (KFN, updated in 2016); [44, 45]	Entrance to practice as pharmacists	Graduate pharmacists must undertake the Competency Examination comprising a Computer Based Test (CBT) followed by an Objective Structured Clinical Examination (OSCE). The exam assesses pharmacists’ knowledge, cognitive skills and professional, legal and ethical decision-making. An alternative was given for pharmacists who graduated before 2011 who did not have a certificate of competence to undertake the OSCE (for pharmacists working in community pharmacy and hospital) or OSPE (Objective Structured Pharmaceutical Examination) for pharmacists working in pharmaceutical industries and wholesalers.

^a^Ikatan Apoteker Indonesia; ^b^Jaminan Kesehatan Nasional = Universal healthcare coverage program; ^c^Satuan Kredit Partisipasi; ^d^Cara Belajar Insan Aktif; ^e^Surat Tanda Registrasi Apoteker; ^f^Komite Farmasi Nasional; ^g^Surat Ijin Praktek Apoteker

### Stakeholders’ interview

29 key stakeholders took part in the interviews between February and August 2016. Characteristics of the interview participants are provided in Table 2.

**Table 2:** Characteristics of Participants

Characteristics	*n* (Total 29)
Male	18
Educational background	
Pharmacists	25
Non-pharmacists	4
Professional background	
Practicing pharmacists	10
Other health care professionals	1
Academics and researchers	4
Pharmacy managers	3
Policy makers and administrators	8
Consumer Representatives	1
Insurance providers	2
Province	
Greater Jakarta	8
Yogyakarta	6
East Java	14
Central Sulawesi	1
Metropolitan/Urban City	23
Method of interview	
Face to face	25
Over the phone	4
Average duration of interview (min)	77 min (range 35–116 min)

Three main themes emerged in the analysis of the data: barriers to effective implementation approaches, expectation for policy changes and coping strategies for the challenges of existing initiatives. Illustrative quotes of the findings are described in Table 3.

**Table 3:** Illustrative quotes of the findings

Topic	Quotes
Challenges to policy implementation	*“When we look at policy changes there are too many hands involved…as you go every layer decisions get diluted, accountability gets diluted, execution gets diluted so there is no strong line for accountability. Who is truly accountable for change of healthcare in Indonesia? Is it the Ministry of health or the police in the region? and you also have very regional influences. You have the region of governance*” (P018_FNP). Lack of accountability in the implementation *“We do have policies, standards, regulations on one hand but on the other hand…we see with our eyes that there is no pharmacist (in pharmacy)…There is no one who pushes the policy, facilitates the policy which means that there is a lack of facilitation especially from government to ensure that the policies are running well. They don’t support it so it is up to pharmacy…”* (P029_MP). Lack of facilitation from government *“We’re only undertaking CPD because we have no choice, it’s not because we want to improve our competence. It’s just because we have the awareness that (collecting) SKP is a prerequisite to continue practicing pharmacy. That is why CPDs and seminars are being treated like reunions…Whether they (pharmacists) practice is another matter. They say, I get SKP so I can extend my STRA (registration), I need STRA to get my SIPA (license), and no SIPA means no salary. Whether I show up for work is my business with my employer; IAI should mind their own business”* (P01_FP). Skeptical to the impact of the policies *“We always look for scapegoats when we do something wrong…The popular excuse when committing violations has been “I can do this because others have done the same and they don’t get punished”. When violations go unpunished, people end up considering these violations as normal”* (P02_FP). Lack of enforcement for successful policy implementation *“Many pharmacists from [name of government bodies] work in pharmacy. I ask them to quit but it is difficult to ask people to become good role models in Indonesia…I ask them to be consistent, consistent with their own policies (they created). It is really shameful if individuals from [name of government bodies] should have been present in the pharmacy three times in a week but it turns out he comes only once in every three weeks. It is embarrassing”* (P027_FP). *“[name of professional organization] cannot become agents of change because there are many people with various interests in [name of professional organization]. There are people who have interests in obtaining official appointments* e.g. *becoming a commissioner for a state-owned enterprise, or director for state owned enterprise. Therefore, it is difficult.” (P028_MP)*. Lack of trust in pharmacy stakeholders
Need for policy changes	*“We were challenged by MoH when we had a coordination meeting. They said “If you could show us the evidence of what can pharmacists do when they practice then we can discuss about their fees”. To date, we are unable to show this evidence”* (P05_MP). *“We can’t use the word evidence at the moment because we (pharmacists) don’t practice, am I correct? The number of practicing pharmacists is very low…Nowadays, they (pharmacists) only talk about business or sales” (P015_MP)*. Collection of evidence *“We try to look for role models. For instance, IAI [name of region] covers five branches and I asked each branch to look for a community pharmacy which can be role model. Then we can replicate the success to other pharmacies, one becomes two, three and so on”* (P027_FP). Search for pharmacy role model *“There is a wide discrepancy between education and practice because universities are still polyvalent (of knowledge)…Frankly speaking, the education system does not create pharmacists to be pharmacists. The education system is overloaded with too many science courses…there is no practice values within the course”* (P015_MP). Changing pharmacy education curricula *“I think they should have collective responsibility but right now they don’t talk each other. So, the universities don’t exactly know where they want to take healthcare to the next stage. The government policy does not have support what comes out and then the IAI also just kind of, I think they are great in showing best practices but not again not execution. I think there is a little bit of within any political maneuvering there are the egos, who should be responsible? which other parties should be responsible for?”* (P018_FP). Lack of a shared stakeholder vision *“(we need) policy that makes pharmacists proud of working in pharmacy, policy that supports pharmacy as the first point of contact with patients, policy that makes pharmacy is a setting to listen to patient’s problem related to medication. That’s all. It is a great thing if we have those three policies”* (P028_MP). Policy advocating pharmacists
Coping strategies initiated by locals	*“we have accreditation system by giving pharmacy star rating from 4 to 1 star…the accreditation evaluates the workforces, facilities, legality for practice, service provision and administrative matters. We give different score for each aspect with service provision is the highest…we do it once in every one or two year and we publish the results regularly…the stars must be displayed in the pharmacy”* (P016_MP). Pharmacy star rating model - applied in Yogyakarta *“When a pharmacist wants to open pharmacy and they have difficulty in purchasing, I offer them my stock at a cheap price. I don’t take profit. That is to push pharmacist practice. When there is pharmacist who opens a pharmacy, I endorse colleague to guide the pharmacist from the scratch, help them with how to provide good service and even they are not yet sustainable for procurement, they can buy to another pharmacy”* (P05_MP). Peer support and assistance - applied in East Java *“If pharmacist is unable to order medicine such as Imodium (brand name of Loperamide) because the price of one tablet is 6 thousand rupiah (approximately 60 cents)…your pharmacy can buy from me. What important is you have the stock of the medicine. We make a network so we can help other small pharmacies. Other cases, for example your pharmacy can’t sell a medicine. By having network you can distribute it to other pharmacies which may be able to sell it. We can help each other so we can minimize loss due to expired medicines”* (P027_FP). Networking and collective approach - applied in Greater Jakarta *“The head of IAI must be strong character person, with vision and knowledge and a resolve to enforce the regulations…It really depends on the leadership, that’s why he should be above any matters involving conflict of interest.”* (P025_MP). Leadership influencer and support - applied in Central Sulawesi *“We have a quality assurance division to ensure pharmaceutical services are correctly delivered. We have many tools for supervising and reporting whether services are correctly provided or not…Home care needs to be done once a week, and every week 5 Patient Medication Records (PMR) need to be filled out…we have records of how many hours spent for patient consultations…we learn something new every time, we have an update training every 3 months minimum. Our skills are up to date, the system is good”* (P01_FP). System of quality assurance - applied in Chain Pharmacy

#### Barriers to effective implementation

Several barriers to implementation approaches were identified by the participants. One commonly expressed barrier is the lack of enforcement. Participants believed the approaches were created with good intention, yet the practice was not strongly encouraged or enforced. When there is a discrepancy between policies and practice, no firm response has been taken by the authorities to discipline the poor practice. The poor enforcement is also associated with a lack of trust in the integrity of the regulatory and supervisory bodies, since individuals in these institutions who violate the policies have not been sanctioned for their misconduct. However, on the other hand, professional pharmacy organizations have also been subject to criticism and participants expressed mixed responses towards them. Several participants criticized them as being unable to advocate the interest of community pharmacists, and instead seeking their own power and financial gain. Others mentioned that the establishment of peer groups within the professional organisation is useful although they are still limited in their influence.

One participant who worked for a multi-national chain pharmacy company expressed her concern regarding the lack of accountability around policy implementation. The operation of community pharmacy has been influenced by a number of policies created and supervised by several different organisations. While community pharmacy has traditionally operated under a highly-regulated environment, the involvement of additional organisations in the monitoring and execution of the policies has increased the complexity and diluted the impact of policy decisions. In addition, government which should play a role in facilitating policy implementation has been viewed by some participants as being lacking in the power to play such role. They highlighted several policies which have been created but not enforced, due to a lack of empowerment and support for the practice.

Skepticism about the impact of these approaches was also expressed by the participants in relation to the imperative for pharmacists to participate in the CPD program. One respondent suggested that participation in CPD is merely seen as a way to collect the required credits (SKP) and not as a means to improve or develop as a professional. She referred to the opinion of some pharmacists who viewed CPD as a gathering or reunion of colleagues and peers. They attended CPD to gain sufficient SKP to renew their license without thinking about the essence of CPD to develop pharmacists’ knowledge.

#### Need for policy changes

While respondents acknowledged that several policies were still ongoing, there was a general consensus that overall insufficient progress has been made. Therefore, they expressed a need for further changes to improve the situation. One major need is to have a unified vision of stakeholders in pharmacy. One participant highlighted the need for collective responsibility to create a vision for the improvement of community pharmacy practice in Indonesia. She urged key stakeholders such as universities, governments and IAI to sit around a table together and create a plan for the advancement of pharmacists. Other participants argued that collection of evidence in community pharmacy is necessary as it is a means to showcase pharmacists’ contribution to the healthcare system. One participant regretted the fact that no evidence can be provided to show pharmacists’ impact. However, another participant perceived that it is impossible to collect evidence as only a few pharmacists practice regularly. Therefore, some participants highlighted the need to duplicate good practice in some pharmacies and amplify it into a policy action. These participants argued that community pharmacy is lacking good role models, and therefore policy supporting the dissemination of good model practice is required.

The majority of respondents anticipated the need for major changes in the pharmacy curricula which are currently still focused on the pharmaceutical sciences. Participants considered that pharmacists are not ready to interact with the patients as they are trained predominantly in laboratory work, and lack exposure to practical and clinical experience. This was linked to poor pharmacists’ attendance in the pharmacy. Some participants highlighted the need for a supportive policy that is intended to make pharmacists and pharmacy as a first point of contact and venue for resolving patients’ problem with medication.

#### Strategies initiated by locals to cope with existing challenges

Despite a number of centrally administered approaches designed to regulate community pharmacy practice, interestingly, some respondents mentioned several local initiatives, led by individuals or local associations, independent of the government and national organization agendas. They claimed that these programs were able to support the role development of community pharmacists, and aimed to increase pharmacists’ participation and presence in community pharmacy.

For instance, the IAI and local health office in Yogyakarta have implemented a star rating system to measure the quality and performance of a community pharmacy. Community pharmacies with the best performance are awarded 4 stars, with the lowest receiving 1 star. This was perceived as an incentive for community pharmacies to increase their performance.

Pharmacy leaders in some regions of East Java have encouraged new pharmacist graduates to open a pharmacy by offering assistance in the procurement of medicines and management of pharmacy. Participants viewed such support as essential to help new pharmacists start professional practice and become competent in the business of pharmacy. Some pharmacy leaders in Jakarta have developed a collective network to help pharmacists in managing their medicines stocks. They also use the network to empower each other, thus pharmacists have a channel to communicate about their practice. In a region outside Java, the leaders of IAI used an interpersonal approach and their leadership to motivate pharmacists to practice while advocating the interests of community pharmacists. In this way, participants expressed that pharmacists are much confident and feel secure as they know that they are supported by their leaders. In addition, participants mentioned the importance of a leader in pharmacy to become a role model for their colleagues, and avoiding unscrupulous and collusive practice.

One participant representing a chain pharmacy business used a quality assurance system to maintain the quality of pharmacy services delivered in her pharmacy and to improve the skills and knowledge of employee pharmacists.

## Discussion

To the best of our knowledge, this is the first study which has collated and evaluated the multiple initiatives designed to influence community pharmacy practice in the context of a developing country. The detailed overview of the major approaches that have been implemented to improve the practice of community pharmacy and pharmacists in Indonesia presents important data to inform the development of future intervention strategies to effect practice change. The findings also contribute to an understanding of policy development and implementation in the Indonesian community pharmacy sector which is currently lacking in the literature.

In collating and summarizing recent policy and other initiatives, it has become apparent that the multiple approaches and regulations introduced into the Indonesian community pharmacy sector over the past ten years reflect an enthusiasm by both policy makers and pharmacy stakeholders to support pharmacists’ role development. Encouragingly, the broad range of the approaches have also demonstrated a significant level of commitment by both groups to improving the current practice in community pharmacy, and a clear recognition of the untapped capacity and potential for community pharmacy to make a greater contribution to the healthcare system overall. This is particularly important in relation to a developing country such as Indonesia where there is limited acknowledgement of the pharmacy profession and the role of community pharmacy in health care as reflected in the low levels of effort to support practice change [21].

Our findings demonstrate that a clear legal framework exists for the regulation and enforcement of the practice of community pharmacy and pharmacists, specifically through the Pharmacy Practice Act and Community Pharmacy Decree, which define the core domain of pharmacy practice which is specific and unique to pharmacists’ role, expertise and authority. Further legislation reinforces the set of skills required to be mastered by community pharmacists as outlined in the Competency Standards. These may then be translated into a range of pharmacy services as regulated by the Standard of Pharmacy Practice and Standard of pharmacy services. Thus, a template for potential practice change and development is present in a formal and supposedly enforceable sense; however a lack of prior supporting research evidence made it very unclear how well (or if at all) these approaches have achieved their policy objectives or adequately addressed the needs of the profession. Our qualitative study was designed to begin to address this gap.

In relation to the second objective, this study reflects the expressed opinions and attitudes of a sample of stakeholders in Indonesia and has provided an insight into the implementation of multiple approaches to advance pharmacy practice as advocated by government and pharmacy professional organization. Seven specific findings are discussed in this section.

Firstly, this study has highlighted that a number of the initiatives, while relevant and appropriate in themselves, were conceived and enacted in a piecemeal, sometimes conflicting and uncoordinated way. For example, the requirement for pharmacists to undertake the CPD program has not been effective in achieving its policy objective. While it is widely believed that CPD is an effective avenue for improving pharmacists’ competency by targeting both educational and experiential learning for participants, our findings suggest that the lessons from CPD among Indonesian pharmacists have not been translated into practice. As expressed by respondents in this study, even mandatory participation in CPD has not been a pathway for improving practice. It is viewed as a way to accumulate a certain number of the credits required to maintain licensure. Moreover, the CPD activities which are available are knowledge-based rather than skills-based or practice-focused and do not necessarily correlate with the pharmacist’s scope of practice. For example, pharmacists working in pharmaceutical industry are able to undertake CPD on the management of hypertension focusing on clinical knowledge more suited to practicing pharmacists in the hospital or community pharmacy setting.

Lack of coordination is also seen in the attempt to set minimum remuneration rates by some local branches of IAI, rather than by the association at the national level. Whilst the reason for the absence of similar initiatives at the national level is unknown, it reflects a lack of consensus regarding minimum remuneration for community pharmacists. Similarly, the initiatives to improve recognition of pharmacists’ role in health care are undermined by the continuing poor level of attendance of pharmacists in many community pharmacies [22]. This latter finding also highlights the lack of enforcement of legislation, which is the second major finding from this study. There are two factors contributing to the lack of enforcement as expressed by participants. Firstly, there is a strong perception that pharmacists who have violated the law will go unsanctioned. Secondly, this perception is reinforced by the observation that some pharmacists working in government authorities whose responsibility it is to enforce the regulation, have also been guilty of its violation and not been sanctioned. Apparently, these two factors – misuse in practice and abuse of power – have not been addressed by current legislative frameworks which demotivates pharmacists from being present in the pharmacy. Whilst recognition of this issue has been put forth in the conception of some approaches such as indicated in the collection of credits (SKP) which includes evaluation for regular attendance, progress is still far from sufficient. The approach of the Chairman of BPOM through a directive prohibiting BPOM staff from working in community pharmacy as mentioned in the introduction of this study might go some way towards redressing the problem.

Thirdly, this study has highlighted a number of key attitudinal barriers to the implementation of practice change approaches, notably strong perceptions of poor policy enforcement, lack of trust in the role of the governing bodies and skepticism towards the impact of the programs. These barriers are not uncommon in developing countries [11, 12], and therefore there is a need to address all these issues - which are notoriously difficult to change - in order to create sustainable and successful policy implementation influencing practice change.

Relatedly and fourthly, this study highlights the desire of stakeholders for a shared vision describing best practice in Indonesian community pharmacy. The commitment to a shared vision ranging from individuals, group of individuals to organisations is essential to overcome the preceding barriers. In addition, the presence of a shared vision particularly between peak pharmacy organisations and the government as regulator will facilitate the development of a role model of community pharmacy and support the collection of evidence through research in community pharmacy. In many developed countries, a shared vision has become common sense for stakeholders in the community pharmacy sector to build a mutual understanding of the future of community pharmacy practice [9, 10, 23].

Fifthly, and accordingly, there was a need to design strategies that can be successfully and sustainably implemented in the setting of community pharmacy. However, with top-down approaches, there also needs to be a recognition that programs with good policy objectives may result in unintended and unwanted consequences. Our previous study analyzing the contemporary situation in community pharmacy in Australia highlighted that knowing the problem or understanding the mechanism to resolve the problem does not guarantee good implementation of a policy. This is particularly because community pharmacy operates in a complex and dynamic system with several key elements from social, policy and economy context influencing the micro (individual pharmacists), meso (community pharmacy as an institution and network of institution) and macro level (healthcare system) of community pharmacy [24]. With respect to Indonesia, community pharmacy continues to face a number of underlying issues such as a shortage of pharmacists, limited clinical competency of pharmacy staff, counterfeit drugs and illegal supply of medicines available from street vendors to healthcare professionals, which is consistent with the situation in many other developing countries [25]. As community pharmacy and the health system are inter-connected, the impact of poorly implemented programs in community pharmacy sector may undermine policy initiatives and create poor outcomes elsewhere in the health system, and therefore policy makers and stakeholders in pharmacy must look at broader scope of the program. This study reinforced the argument that simply adding new policies or strategies will not improve the situation without resolving the underlying problems of the past.

Sixthly, the findings of this study also highlight the potential feasibility of a national scale-up of local interventions. The successful local initiatives described by participants illustrate a range of novel and different ways to enhance pharmacists’ roles, tailored to the specific context in which they operate. In addition, these initiatives reflect a desire and willingness of local organisations to address the challenges of policies designed at the national level. Expanding this bottom up approach will undoubtedly require a good understanding of local situations and may be unique in every region. However, some key characteristics of successful approaches have emerged. Most of the local initiatives included in this paper involved a collective approach through networking and mentoring to encourage pharmacists to practice. Others relied on the critical role of leaders in recognising the need to support and encourage individual pharmacists. A few initiatives involved the application of a quality assurance system by ensuring adequate resources for pharmacy operations and maintaining the quality of the services by implementing pharmacy rating star model as a showcase for consumers and patients. While these local strategies were not supported by robust evidence of effectiveness, they may act as catalysts for change whereby local pharmacists’ communities work together for a common purpose and for better results. Another lesson is that sustainable changes are often achieved through an understanding of local health care needs. The UK experience in introducing the Healthy Living Pharmacy program which allows an individual pharmacy to tailor services to local needs despite the pharmacy being contracted under the NHS scheme is an example [26]. This is also similar in Australia where a number of pharmacists under Health Destination Pharmacy program have changed their practice by adopting a bottom up approach where they can be innovative and adapt to the changing demand in the current state of healthcare [27].

Finally, and underpinning all aspects of practice change, is the urgent need to transform the current pharmacy education system which has hitherto primarily focused on pharmaceutical sciences rather than on pharmacy practice. In responding to the changes in pharmacy which focus on the role of pharmacists in medication management and patient centered care, the education system in Indonesia - which consists of four-years undergraduate and one-year apothecary program - must be reshaped to better prepare pharmacists, not only for future changes, but also for the current situation. Reflecting on our findings, it is imperative to devote more time, effort and resources to developing the clinical knowledge and experience of pharmacy students to allow them to face and meet the challenges in healthcare that they will experience during their careers.

The interpretation of the study results should take into consideration a number of limitations. Firstly, the assessment of the policy or program was limited to reporting experience and perception of stakeholders with no quantitative measurement showing quality performance of the policies. While providing such data is also of importance, we were interested to capture the key aspects of the lived experience of program functioning and its impact on community pharmacy. Secondly, the study only identified the approaches delivered by national organizations or central government authorities without including assessment of policy at the lower bureaucratic level such as policies created by local government. Hence, underreporting of strategies within this paper is possible. We did not attempt to collect data on the policies or programs at the lower level although they are likely to influence community pharmacy. One of the reasons was due to the scarcity of available information about the policies. However, assessing the national policy agenda has enabled an overarching understanding of the broad spectrum of initiatives that have been undertaken to improve community pharmacy practice in Indonesia. Thirdly, it might be argued that each individual study participant would be expected to have only a relatively narrow and specialized understanding or experience of the Indonesian health care/pharmacy system, however the number and diversity of the participants meant that a wide range of perspectives was obtained. In fact, the breadth of the stakeholder cohort was a strength of this study since the policies of interest could be interrogated from multiple angles. Finally, new policies have been introduced following the research underpinning this paper, for example, the implementation of a policy allowing pharmacists to work in a maximum of three different pharmacies as regulated under the Decree for registration, certification and licensure of pharmacists. On the surface, it seems unlikely that that this policy will have a significant impact on the practice in community pharmacy, however, it is important to keep tracking such changes to see the impact in the future.

## Conclusion

The introduction of a plethora of policies, regulations and initiatives within the past ten years has highlighted the enthusiasm of policy makers and pharmacy stakeholders to improve community pharmacy practice in Indonesia. However, some of the initiatives were conceived and enacted in a piecemeal, sometimes conflicting and uncoordinated way. Despite the good policy objectives of the initiatives, it appears that poor enforcement, lack of trust of pharmacy stakeholders and skepticism regarding the impact of the initiatives have significantly undermined the success of these initiatives, and remain the predominant challenges for successful policy implementation. This study suggested some attempts to resolve these challenges focusing on the need to have a shared vision among peak pharmacy stakeholders defining best practice in community pharmacy. Some local initiatives highlighted the bottom-up approach in the system and potential for scaling up at the national level. Overall, it is clear that some fundamental and entrenched barriers to practice will need to be overcome in order to create a more professional climate for the practice of pharmacy in Indonesia.

## Additional file

Additional file 1: Interview guide. (DOCX 13 kb)

## Abbreviations

APTFI: Assosiasi Perguruan Tinggi Farmasi Indonesia (Indonesian Association of School of Pharmacy)
BPOM: Badan Pengawas Obat dan Makanan (Indonesian National Agency of Drug and Food Control)
CBIA: Cara Belajar Insan Aktif (Active Individual Learning)
CPD: Continuing Professional Development
DAGUSIBU: Dapatkan, Gunakan, Simpan, Buang Obat (Campaign on self-management of medicines)
GEMA CERMAT: Gerakan Masyarakat Cerdas Menggunakan Obat (Community Awareness Campaign in using Medicines)
GKSO: Gerakan Keluarga Sadar Obat (Campaign for raising Family Awareness in using Medicines)
GP Farmasi: Gabungan Perusahaan Farmasi Indonesia (Indonesian Pharmaceutical Association)
HISFARMA: Himpunan Seminat Farmasi Masyarakat (Community Pharmacists Group)
IAI: Ikatan Apoteker Indonesia (Indonesian Pharmacists Association)
JKN: Jaminan Kesehatan Nasional (Universal Healthcare Coverage)
KFN: Komite Farmasi Nasional (National Pharmacy Board)
MoH: Ministry of Health
PRISMA: Preferred Reporting Items for Systematic Reviews and Meta-Analyses
SKP: Satuan Kredit Partisipasi (credits for participation)
STRA: Surat Tanda Registrasi Apoteker (Pharmacist Registration Letter)
