# Supplementary material for: Multiple policy approaches in improving community pharmacy practice: the case in Indonesia
Source: BMC Health Serv Res. 2018 Jun 14;18:449. doi: 10.1186/s12913-018-3258-8 (PMC6001118; doi:10.1186/s12913-018-3258-8)
Supplement: Supplementary file 1 — Interview guide. (DOCX 13 kb) [file 12913_2018_3258_MOESM1_ESM.docx]

**Interview guide**

1. How do you view the current situation in Indonesian Community Pharmacy?
2. Do you acknowledge some changes in the landscape of community pharmacy sector?

*Policy framework in community pharmacy*

1. What do you think is the main policy framework that underpins the contemporary practice of community pharmacy?
   1. How influential are the policies or regulations for supporting contemporary practice?
   2. Have they been enforced adequately? Who does hold the authority for enforcement?
   3. Are there any other policies influencing the practice of community pharmacy?
2. What do you think are the challenges for implementing the policies as you mentioned?
   1. Are these barriers common to pharmacy practices? Yes, are they pertinent issues? No, please explain?
   2. Why do you think these barriers occur? Can they be prevented?
3. What is your effort to cope with the challenges presented by the policy changes?
   1. Do other pharmacies or pharmacists have the same ideas as yours? **If Yes**, please explain? **If No**, why others don’t?
   2. How applicable do you think your solution for other pharmacies/pharmacists?
4. Given the changing nature of pharmacy, do you see any need for establishing new policies to improve the practice of community pharmacy? **If Yes**, why and what are the policies needed? **If No**, why not?
5. What is your expectation to improve the current practice of community pharmacy? who should be responsible for that and what is your suggestion for them?
